# Examining the Effectiveness and Efficiency of an Innovative Achievement Goal Measurement for Preschoolers

**DOI:** 10.3389/fpsyg.2021.741088

**Published:** 2022-01-05

**Authors:** Chung Chin Wu

**Affiliations:** Department of Early Childhood Education, National Pingtung University, Pingtung, Taiwan

**Keywords:** achievement goals, effectiveness, efficiency, measurement invariance, preschoolers

## Abstract

A large number of studies have investigated achievement goals and their related antecedents and consequences above elementary school level. However, few studies have implemented achievement goal assessment to investigate achievement goals and their relevance for preschoolers. In particular, no valid measurement has been developed for preschoolers’ self-reporting of their achievement goals. The main purposes of this study were twofold: (1) To develop an innovative achievement goal measurement for preschoolers, and to investigate the best theoretical model for understanding preschoolers’ achievement goal across gender. (2) To examine the effectiveness and efficiency of the pictorial and pure text measurement format and approaches (for young children’s self-reporting and teachers’ rating purposes, respectively). A total of 364 preschoolers aged 5 years participated in self-report activity, and 32 preschool teachers obtained consent to rate 193 out of 364 preschoolers. Results showed: (1) the developed achievement goal measurement was a valid tool for understanding preschoolers’ achievement goals and was equally suitable for boys and girls. (2) The 6-factor achievement goal model was the best theoretical perspective for understanding preschoolers’ achievement goals for both boys and girls. (3) The pictorial measurement format for preschoolers’ self-reporting of achievement goals was a more effective but less efficient way to investigate preschoolers’ achievement goals, while the opposite was the case for the pure text measurement format for teachers’ ratings. Implications for achievement goal literature and future research are discussed.

## Introduction

Competence is the conceptual centerpiece of achievement and achievement motivation (Elliot and Dweck, 2005). Competence is defined as a condition or quality of effectiveness, ability, sufficiency, or success (Elliot et al., 2017). For example, preschoolers may feel competent if they are able to complete a task successfully by themselves, their ability of doing things independently is demonstrated and they experienced success. Evaluation of competence not only affects academic achievement but also the formation of personality (i.e., academic self-concept; Arens et al., 2011; Hughes et al., 2011). In achievement motivation literature, achievement goal theory is specifically proposed to address how competence is evaluated. It has been demonstrated that there may be positive or negative consequences when different referents (i.e., the requirements of the task itself or others’ performance) are used to evaluate one’s own competence. Therefore, individuals may be motivated to approach desirable outcomes (i.e., mastery of task or having superior performance to others) or avoid undesirable outcomes (i.e., unable to master task or having an inferior performance to others). For example, devoting time to task mastery may lead to greater class interest (Harackiewicz et al., 1997), while being afraid of being unable to master a learning task may cause disorganized approach to study (Elliot and McGregor, 2001). It is beneficial to have an understanding of the achievement goal before commencing task participation; this enables appropriate instruction intervention to adopted prior to the task beginning to promote or maintain the adaptive goal (i.e., dedicate time to mastery learning task). This prevents the adaptive goal and the maladaptive goal (i.e., try to avoid doing worse than other), respectively, from declining and increasing over time (Anderman and Midgley, 1997; Anderman et al., 1999; Meece and Miller, 2001; Bråten and Olaussen, 2005; Fryer and Elliot, 2007; Shim et al., 2008), and further affects learning performance (Lin and Hsieh, 2000; Lau and Nie, 2008; Daeun et al., 2016).

Achievement goal theorists have operationalized achievement goals into different dimensions (i.e., 2-, 3-, 4-, or 6-factor models; Pintrich, 2000; Elliot et al., 2011). Taking different perspectives and approaches, many studies have focused on investigating achievement goals and their antecedents, and together with main consequences for students above elementary education level; most of these studies have used a self-report method for data collection. However, few investigations have been conducted at preschool level. Although a few researchers attempted to measure preschoolers’ achievement goals through researchers’ ratings based on a presumed achievement goal framework (i.e., 2-factor model) and experimental manipulations (Smiley and Dweek, 1994; Butler, 1996; Butler, 1998), it was not possible to qualitatively determine the accuracy and effectiveness of achievement goal measurement by others because the evidence for construct validity were absent. Furthermore, the extent to which experimental manipulation and rating by others measures what it is intended to measure is rarely subject to debate.

Measuring achievement goals through preschoolers’ self-reporting can provide valid qualitative evidence. However, no suitable achievement goal measurement has yet been developed for preschoolers to self-report their achievement goals. This may be because preschoolers’ cognitive evaluation ability was underestimated for a long period of time. Consequently, it remained unclear which is the best theoretical model for understanding preschoolers’ achievement goals, and whether or not preschoolers could self-report their achievement goals. Picture books are widely favored by preschoolers, and researchers used this format for a long time to assess the receptive language and vocabulary of 3- to 6-year-old children (i.e., Peabody Picture Vocabulary Test; Dunn and Dunn, 2007; van Rootselaar et al., 2021). Hence, the utility of this pictorial format for measuring preschoolers’ achievement goals was demonstrated. The pictorial format can be adopted to enable self-reporting by preschoolers, while the pure text format can be adopted for rating by others; the two formats should hence be compared to demonstrate the effectiveness and efficiency of both approaches for investigating preschoolers’ achievement goals.

The purposes of this study are twofold:

1. To develop an instrument for measuring preschooler’s achievement goals, and to determine the best theoretical model for understanding their achievement goals across gender.
2. To examine the effectiveness and efficiency of two measurement formats and approaches: self-reporting by preschoolers and rating by others.

## Development of Achievement Goal Theory

Over the past four decades in the achievement motivation literature, a majority of empirical research studies have adopted the achievement goal perspective. Achievement goals represent the reasons for achievement behaviors and people’s cognitive-dynamic focus while engaged in achievement-related behaviors (Ames, 1992; Urdan, 1997; Elliot and McGregor, 2001). Theorists initially proposed two distinct goals for achievement behaviors: task involvement vs. ego involvement (Stipek, 1984) and learning goal vs. performance goal (Dweck, 1986). The definitions produced were similar enough to be integrated to form a dichotomous (2-factor) achievement goal model consisting of a mastery goal and performance goal. The mastery goal leads individuals to focus on mastery of the task and competence and is defined by means of intrapersonal or absolute standards, while the performance goal orients individuals to relate their performance to that of others (thereby focusing on normative standards; Pintrich, 2000; Wirthwein et al., 2012).

A 3-factor model was later proposed, containing the same mastery goal but bifurcating the performance goal based on approach and avoidance predispositions to form a performance-approach goal and performance-avoidance goal (Elliot, 1999). Later, the 4-factor achievement goal model was bifurcating the mastery goal based on approach and avoidance predispositions; the performance-based goals in the 3-factor model were held constant in this model (Elliot and McGregor, 2001). As a result, four factors were included in this model: mastery-approach, mastery-avoidance, performance-approach, and performance-avoidance goals.

Elliot et al. (2011) further re-conceptualized mastery-based goals (mastery-approach and mastery-avoidance goals) on the basis of two referents from the 4-factor model: absolute task standard and intrapersonal standard. Accordingly, a 6-factor achievement goal model was proposed which encompassed task-approach, task-avoidance, self-approach, self-avoidance, other-approach, and other-avoidance goals. Task-based goals (task-approach and task-avoidance goals) define competence in terms of achieving or not achieving the task requirements. Self-based goals (self-approach and self-avoidance goals) identify competence in terms of performing well or poorly in comparison to one’s past performance. Other-based goals (other-approach and other-avoidance goals) take an interpersonal standard as a referent to evaluate one’s own competence relative to others’ performance. Approach and avoidance predispositions guide someone to approach the positive (achieving the task requirements or performing well) and avoid the negative (not achieving the task requirements or performing poorly) possibilities, respectively.

### Supporting Evidence for Achievement Goal Models

Huang (2012) conducted a meta-analysis synthesizing 151 studies to compare the validity of the 2-, 3-, and 4-factor achievement goal models. The results indicated that the 4-factor achievement goal model was the best choice for understanding the learning outcomes of students above elementary school level. About the same time as Huang’s research was completed, a new theoretical model was proposed to re-conceptualize the 4-factor model into a 6-factor achievement goal model. Elliot et al. (2011) compared several alternative models (i.e., 2-, 3-, and 4-factor models) with their 6-factor theoretical model. The findings showed that the 6-factor achievement goal model was the most suitable for understanding undergraduates’ achievement motivation. The 6-factor model was then applied to elementary school high-graders, junior high school (Wu, 2014), senior high school (Méndez-Giménez et al., 2017), undergraduate (Elliot et al., 2011, 2015), and adult samples (Mascret et al., 2017). In summary, there was supportive evidence for both 4- and 6-factor achievement goal models; empirical evidence has accumulated in favor of the 6-factor model. However, it remains unclear which is the best theoretical model for understanding preschoolers’ achievement goal.

### Need for a Valid Way to Investigate Preschoolers’ Achievement Goals

The achievement goal literature is dominated by quantitative research. Questionnaires were the most commonly used method to investigate subjects’ achievement goals and related issues, whereas experiments are used in relatively small number of studies (for an overview, see Hulleman et al., 2010; Huang, 2012). In these studies, subjects older than 8–9 years are assumed to be able to provide self-report information on the items under investigation. Preschoolers’ achievement goals have rarely been investigated, with two exceptions. Butler (1996) introduced manipulated mastery and performance goal conditions to investigate the effects of 5-year-old preschoolers’ achievement goals on their motives for attending to peers’ work. Smiley and Dweek (1994) used four puzzle tasks (composed of insolvable and solvable tasks) asking 4- and 5-year-old preschoolers to decide which of the four puzzles they would like to work on again and to give reasons for their choice. Preschoolers’ preference for working on an insolvable or solvable puzzle followed by an experience of failure and the reasons for their choice were used as indicators of mastery or performance goals. Achievement goals were then classified according to preschoolers’ preferences to predict emotional and cognitive outcomes. The results highlighted the very early emergence of achievement goals. However, of the available studies, only one experimental study attempted to categorize preschoolers’ achievement goals into pre-determined mastery and performance goals. Furthermore, no study attempted to develop a valid instrument to investigate preschoolers’ achievement goals by means of self-report (Berhenke et al., 2011), despite its usefulness for investigating preschoolers’ achievement goals and their large-scale effects on achievement and substantive learning outcomes.

## Hypothesized Achievement Goal Model for Preschoolers

Research has demonstrated that compared to older children, preschoolers are more likely to overcome difficulty to achieve mastery by means of a higher level of endeavor and persistence (Rholes et al., 1980), and they are especially showed more engagement, enjoyment, and persistence on challenging task while they have the opportunity to collaborate with others (Butler and Walton, 2013; Master et al., 2017); they could tell researchers that they were able to build a block tower to a certain height without making mistakes (Smiley and Dweek, 1994). Infants as young as 2 years old tend to turn away when they do poorly on a task (Wigfield et al., 2015). Similarly, Cimpian (2017) observed that young children, aged 4 years, tended to withdraw from the block area after encountering a few difficulties. Recently, preschool with playful context was found to be beneficial for 3- to 5-year-old preschoolers’ mastery motivation (Sawyer, 2017); Israeli 4- to 5-year-old preschoolers were found to show task-approach goal similar to preschoolers in European and displayed lower levels of task-avoidance than European children at the same age (Brody et al., 2018). These suggest that preschoolers may have task-approach and task-avoidance goals.

According to Stipek (1984), preschoolers have the ability to use their past achievement to assess their current performance and that of others’. Stipek and Hoffman (1980) also found that 3-year-old children were already capable of evaluating their current performance through retrieving knowledge of their own past performance on the same task; their expectation of success declined when they perceived their current performance as inferior to their past performance. Furthermore, Stipek and Hoffman also demonstrated that 4-year-old children had already started to judge their own work performance through observation of others; Lapan and Boseovski (2017) proposed a similar finding for young children aged 5 years. By the age of 4 years, children have already started to evaluate their work by taking others as referents or to engage in dialogue related to social comparisons (Mosatche and Bragonier, 1981; Pomerantz et al., 1995). Through experiment and interview methods, researchers found that when 4-year-old children perceived their performance as inferior to others, they tended to avoid engaging in the activity or to have negative feelings (Butler, 1998; Ihmeideh, 2015).

The above findings suggest that preschoolers not only try to define their competence in terms of an absolute standard (requirements of task), an intrapersonal standard (their own past performance), or an interpersonal standard (others’ performance), but that they attempt to approach positive or avoid negative results. This suggests that the 6-factor achievement goal model may be the most suitable for understanding preschoolers’ achievement goals. However, this has not previously been acknowledged and tested in the existing literature.

## Methodology

### Participants

A pilot study was conducted to confirm that preschoolers were capable of understanding the wording in the measurement; the sample consisted of 20 preschoolers aged 5 years. In the formal study, a total of 364 (185 males and 179 females) preschoolers aged 5 years and 32 preschool teachers, selected from 36 preschools in Taiwan, consented to participate in the study. Preschool teachers rated 193 out of 364 preschoolers (93 boys and 100 girls). It should be mentioned that only one teacher was male because male teachers are rarely accepted by parents in Taiwan. This disparity is considered a limitation of this study. Participants were assured that all of their responses would be kept confidential and preschoolers and their parents were informed that their participation would not influence their treatment by the teachers.

### Instruments

Following criteria were set to enable preschoolers to self-report their achievement goals. First, the content used in the measurement had to be familiar to the preschoolers (Bulter, 2005). This was beneficial for reducing cognitive loading and to enhance the capacity and ability of memory retrieval and manipulation (i.e., to retrieve information related to the content of pictorial measurement and to compare the similarity between internal and external information; Anmarkrud et al., 2019). Second, in contrast to questionnaire items for students above elementary level, the measure could not be presented in the form of a text description without accompanying context (for an overview of measuring items, see Hulleman et al., 2010). Third, each item needed to have no more than five response options (Marsh et al., 1991).

To meet the first and second requirements, a qualitative study was conducted to collect information that preschoolers were familiar with; this then formed the content of the measurement items. Specifically, preschoolers’ dialogue and behaviors during their daily school work in the block area were observed and recorded. The block area was chosen because it is the most common learning area in preschools in Taiwan, and the tasks preschoolers were trying to complete in this area were also similar to those used in Smiley and Dweek’s (1994) experimental study. Through 367 observations in the block area, the dialogue and behaviors of several preschoolers related to the hypothesized 6-factor achievement goal model were identified. Several sentences encompassing the observed dialogue and behaviors were formulated, and 21 pictures were developed to represent content in these sentences relating to different achievement goals. The 6-factor achievement goal model was adopted as the framework of this pictorial measurement because the items based on it could be integrated with each other to form alternative models. For example, items for the task- and self-approach goals could be loaded together to measure the mastery-approach goal in the 4-factor model. Each achievement goal was measured by three pictorial items, each represented by a picture (except for three items which had two pictures each because the single statement was difficult to express with single picture). The sentences related to pictorial items were read aloud to each preschooler. Previous research has demonstrated the validity of using pictorial items implemented by reading aloud to young children to obtain preschoolers’ self-reporting data (Paris and Paris, 2003). Four response options, including “very much unlike me,” “a little unlike me,” “a little like me,” and “very much like me,” were designed to preliminarily test whether preschoolers were capable of distinguishing the differences between the options, and to choose the best option to reflect their achievement goal. By doing so, the third of the above criteria was satisfied.

Twenty-one pictorial items were designed to measure the 6-factor achievement goals, each with at least three paired pictures and a brief story description. Pseudonyms were used for character roles in the story descriptions. Sample stories for each of the 6-factor achievement goals for boys were as follows: (1) Bob concentrated on building a castle in the block area (task-approach goal). (2) Bob tells the teacher: “I want to build a castle which is higher than I have made in the past” (self-approach goal). (3) Bob competed with Tom and says: “I want to build a castle higher than yours” (other-approach goal). (4) Bob ran away from the block area because he could not build a castle well (task-avoidance goal). (5) Bob tells Tom: “I do not want to stack up blocks lower than I have made in the past” (self-avoidance goal). (6) Bob competed with Tom, and says: “I do not want to stack up blocks lower than yours” (other-avoidance goal). For girls, the pseudonyms used in the pictorial items were changed to girls’ names. Considering preschoolers’ limited text reading ability, descriptions of each story were read out loud to them.

**Figure 1:**
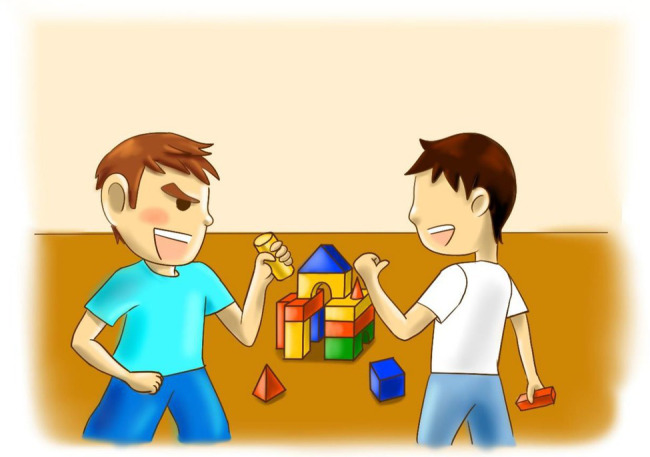
Pictorial achievement goal item for boys to measure other-approach goals.

**Figure 2:**
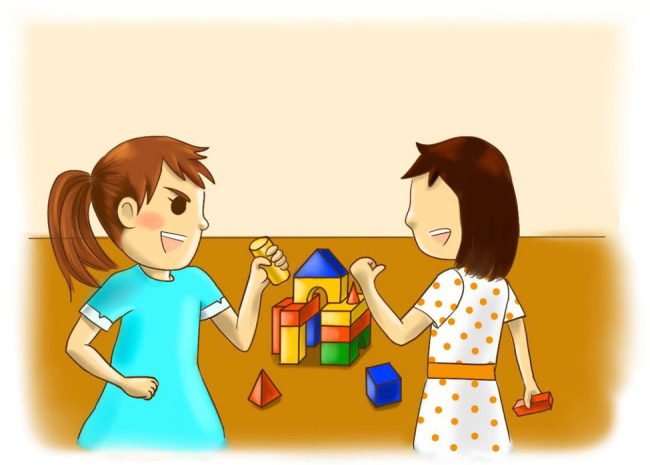
Pictorial achievement goal item for girls to measure other-approach goals.

According to empirical evidence, infants as young as 1.5 year old are already capable of recognizing that the action and psychological states of others are “like them” (Meltzoff, 2007), it suggests that older preschoolers can well evaluate the similarity between the figures in the pictorial measurement and themselves. After each short story was presented, preschoolers were required to answer to what extent the dialogue and/or behaviors of the protagonist in the story was similar to their own. They were required to choose one from the four options on a 1 (“very much unlike me”) to 4 (“very much like me”) scale. Each option was represented by a cartoon face, as used in Smiley and Dweek’s (1994) study. The face scale for rating achievement goals consisted of a row of four yellow schematic faces. The lines representing the mouths ranged from a pronounced downward curve to a pronounced upward curve across the four faces. The left-most face was described as “very much unlike me,” the next as “a little unlike me,” the next as “a little like me,” and the right-most as “very much like me.” Two versions of the measurement were developed, respectively, for boys and girls to prevent the possible effect of consistency between the gender of characters and that of the preschoolers on preschoolers’ responses. For example, boys may report they are very much unlike the protagonist in the story just because the protagonist is a girl. Sample pictorial items for the other-approach goal presented to boys and girls, respectively, are presented in Figures 1, 2. As can be seen, the two versions differed in the hairstyle and clothing of the characters.

Pictorial achievement goal measurement was only used for self-reporting by preschoolers. Pure text descriptions were extracted from those originally used in the pictorial items and was adopted for teacher rating, which is a commonly used format in achievement goal questionnaires. Approximately half of the participating preschoolers were rated by teachers according to the text descriptions. Teachers were requested to evaluate specific young children at a particular time based on their observations and understandings of those children. Teachers rated 93 boys and 100 girls selected from the preschooler participants and had to respond to a total of 18 items (statements).

### Analysis

In order to determine the best theoretical model for understanding preschoolers’ achievement goals, four primary theoretical models encompassing 2-, 3-, 4-, and 6-factor achievement goals were analyzed. The 2-, 3-, and 4-factor models were constructed by rearranging the pictorial items (for preschoolers’ self-reporting) or pure text items (for teachers to rate preschoolers) developed on the basis of the 6-factor model. For the 2-factor achievement goal model, composed of the mastery goal and performance goal, items for measuring task-based and self-based goals were loaded together on the mastery goal, while items for measuring the other-based goal were loaded together on the performance goal. For the 3-factor achievement goal model, composed of the mastery goal, performance-approach goal, and performance-avoidance goal, items for measuring the task-based and self-based goals were loaded together on the mastery goal, while items for measuring the other-approach goal and other-avoidance goal were, respectively, loaded on the performance-approach goal and performance-avoidance goal. For the 4-factor achievement goal model, composed of the mastery-approach goal, mastery-avoidance goal, performance-approach goal, and performance-avoidance goal, items for measuring the task-approach and self-approach goals were loaded together on the mastery-approach goal, while items for measuring the self-approach goal and self-avoidance goals were loaded together on the mastery-avoidance goal, and items for measuring the other-approach goal and other-avoidance goal hold the same as it was in the 3-factor model.

Confirmatory factor analyses (CFAs) were conducted by using MLR (ML estimation with robust standard errors) as estimator, and several indices were used to evaluate the goodness of fit of the four models to the data: the chi-square statistic (*χ*^2^), the comparative fit index (CFI), the Tucker-Lewis index (TLI), the root-mean-square error of approximation (RMSEA), Akaike Information Criterion (AIC), and Bayesian Information Criterion (BIC). The following criteria were used to evaluate the adequacy of model fit: CFI ≥ 0.95, TLI ≥ 0.95, and RMSEA ≤ 0.06 (Hu and Bentler, 1999; Wang and Wang, 2012). CFI and TLI ≥ 0.95, and RMSEA ≤ 0.06 indicated that the model fitted the data very well. 0.90 ≤ CFI < 0.95 and 0.90 ≤ TLI < 0.95, and 0.06 < RMSEA ≤ 0.08 indicated that the model just reached an acceptable level. Δ*χ*^2^, AIC, and BIC were used for model comparisons. Δ*χ*^2^ was obtained by subtracting the *χ*^2^ value of the 6-factor model from that of the alternatives. By comparing the hypothesized 6-factor achievement goal model with the alternative models, a significant Δ*χ*^2^, lower AIC, and lower BIC values indicated the best theoretical model.

After the best model was confirmed, the internal structure of this model was examined. Four indices were adopted: standardized factor loadings, individual item reliability, composite reliability (CR), and average variance extracted (AVE). Convergent validity is achieved if the standardized factor loadings, individual item reliability, CR, and AVE are above 0.71, 0.50, 0.60, and 0.50, respectively (Fornell and Larcker, 1981; Hair et al., 2009). In addition, discriminant validity is preliminarily verified if the 95% confidence intervals of the inter-correlation coefficients among all latent factors calculated by the bootstrap method does not include 1 in the range (Torkzadeh et al., 2003).

After the best theoretical model was determined, a multiple-group CFA was conducted to test the measurement invariance to ensure that the two measurement versions were equally suitable and that the items held the same meaning for boys and girls. Five models were applied to test measurement invariance with the latter models posing increasingly stringent limitations on their parameters. The five models are as follows: (1) configural model: tests whether the two groups are identical in the number and patterns of factors; (2) metric model: examines whether models for different groups hold identical patterns of factors and an invariant factor loading pattern; (3) scalar model: verifies whether models for different groups have identical patterns of factors, invariant factor loadings and intercepts of items; (4) factor variances: examines whether models for different groups hold identical inter-correlations among factors and factor variances; and (5) residual variances: test whether models for different groups hold identical residual variances of items (Cheung and Rensvold, 2002; Wang and Wang, 2012).

The configural model serves as the baseline against which all subsequent tests are measured for comparing equivalence. If metric invariance is violated, the construct may have different meanings for each group. Scalar invariance needs to be achieved in order to compare factor means across gender (Byrne, 2004). CFI and TFI ≥ 0.95, RMSEA ≤ 0.06, ΔCFI ≤ 0.01, and ΔTLI ≤ 0.02 are considered supportive evidence of the more stringent model (Hu and Bentler, 1999; Cheung and Rensvold, 2002). The most stringent model that passed the above criteria was confirmed as the final model, which represents the model with the best level of measurement invariance across gender. In general, scalar invariance was considered as strong measurement invariance and was sufficient for supporting measurement invariance across gender, while the factor and residual variance were too strict to be achieved in practice (Steenkamp and Baumgartner, 1998; Byrne, 2004).

## Results

### Measurement Models of Preschoolers’ Achievement Goals

Table 1 presents the goodness of fit of the hypothesized model (6-factor model) and alternative models and the indices for model comparisons. The results strongly support the hypothesized 6-factor achievement goal model and were obtained through analyzing the data from both preschoolers’ self-reporting and teachers’ ratings. For the preschooler sample, each of the fit indices met the criteria for a good-fitting model: *χ*^2^(120, *N* = 364) = 166.87, *p* < 0.05, CFI = 0.98, TLI = 0.98, RMSEA = 0.033 (90%CI ranged from 0.020 to 0.044). For the teacher sample, the 6-factor model also fitted to the data: *χ*^2^ (120, *N* = 193) = 217.82, *p* < 0.05, CFI = 0.95, TLI = 0.94, RMSEA = 0.065 (90%CI ranged from 0.051 to 0.079). For the preschooler sample, the 6-factor model fitted well to the data: the CFIs and TLIs were all above 0.95, and RMSEA was below 0.06. However, the 6-factor model for the teacher sample fitted to the data only at an acceptable level, because the CFI was equal to 0.95, TLI was below 0.95, and RMSEA was above 0.06.

**Table 1:** Goodness of fit and comparisons of all models.

Model	*χ* ^2^	*df*	CFI	TLI	RMSEA (90% CI)	Δ*χ*^2^	AIC	BIC
**Preschoolers’ self-reporting (**N** = 364)**								
6-factor	166.87	120	0.98	0.98	0.033 (0.020–0.044)		15622.38	15891.28
4-factor	975.05	129	0.71	0.66	0.134 (0.126–0.142)	808.18^*^	16377.14	16610.96
3-factor	1906.32	132	0.40	0.30	0.192 (0.185–0.200)	1739.45^*^	17165.22	17387.36
2-factor	2087.53	134	0.33	0.24	0.200 (0.193–0.208)	1920.66^*^	17496.23	17710.57
**Teachers’ ratings (**N** = 193)**								
6-factor	217.82	120	0.95	0.94	0.065 (0.051–0.079)	–	6818.09	7043.21
4-factor	371.21	129	0.87	0.85	0.099 (0.087–0.110)	153.39^*^	6974.19	7169.95
3-factor	461.71	132	0.83	0.80	0.114 (0.103–0.125)	234.89^*^	7073.40	7259.37
2-factor	1305.66	134	0.39	0.30	0.213 (0.202–0.223)	1087.84^*^	8049.58	8229.02

* *p* < 0.05.

In order to ensure the best achievement goal model, the differences between the chi-square values, the AICs, and the BCCs were used to evaluate the relative fit of the 6-factor model compared with the alternative models. The *Δχ*^2^ values under each degree of freedom were all significant at the 0.05 level. The 6-factor model also demonstrated the lowest AIC and BIC values of all models (AIC = 15622.38/6818.09, BIC = 15891.28/7043.21 for preschooler/teacher samples). This demonstrated that the 6-factor model provided a better fit to the data than any other alternative models for both samples. The 6-factor model appears to be the best model for understanding preschoolers’ achievement goals, but the construct validity of measurement is only well demonstrated in the preschooler sample and not the teacher sample.

### Internal Quality of Measurements

There were two measurement versions which only differed in their format: pictorial and pure text achievement goal measurement for self-reporting by preschoolers and rating by teachers, respectively. The internal quality of measurements was further examined based on the best-fitted theoretical model. For the pictorial achievement goal measurement (based on the 6-factor model) with responses provided by preschoolers themselves, almost all the standardized factor loadings and individual item reliabilities were above 0.71 and 0.50, respectively (except for two standardized factor loadings of 0.68 and 0.69, and individual item reliabilities of 0.46 and 0.48). All the CRs and AVEs were above the criteria of 0.60 and 0.50, respectively. The bootstrap method was conducted, and results showed that the 95% confidence interval of inter-correlations among latent variables ranged from −0.19 to 0.53 (1 was not included in this range). Both convergent validity and discriminant validity of the 6-factor achievement goal model represented by the pictorial format were demonstrated in general.

For pure text measurement (based on the 6-factor model) rated by teachers, there were four standardized factor loadings; their individual item reliabilities were, respectively, below 0.71 (0.70, 0.70, 0.61, 0.57) and 0.50 (0.49, 0.49, 0.37, 0.32), while the remainder were above the criteria (coefficients ranging from 0.74 to 0.91 and 0.55 to 0.83). In addition, all the CRs were above the criteria of 0.60 (ranging from 0.68 to 0.93), and almost all the AVEs were above the criteria of 0.50 (one coefficient was 0.42, with the remainder ranging from 0.56 to 0.81). Bootstrapped results showed that almost all the 95% confidence intervals of the inter-correlations among the latent variables did not include 1 in this range, except for one coefficient which ranged from 0.95 to 1.10. Convergent validity and discriminant validity of the 6-factor achievement goal model represented by the text format were partially supported by the teacher sample.

### Measurement Invariance

Multiple-group CFAs were conducted to examine whether or not the 6-factor achievement goal measurement is invariant across gender. As can be seen in Table 2, for the preschooler sample, all CFIs and TLIs were above the cutoff value of 0.95, and RMSEAs were also below 0.06 in all models. The ΔCFI was ≤0.01 and ΔTLI ≤ 0.02, as calculated by comparing the metric model to the configural model. Similarly, scalar invariance was also achieved because the ΔCFI ≤ 0.01 and ΔTLI ≤ 0.02 when the scalar model was compared to the metric model. However, invariance of factor variances and residual variances failed to be achieved because the ΔCFI > 0.01 and ΔTLI > 0.02 when the factor variances model was compared to the scalar model.

**Table 2:** Measurement invariance tests of the 6-factor model.

Model	*χ* ^2^	*df*	Δ*χ*^2^	RSEAM (90% CI)	CFI	ΔCFI	TLI	ΔTLI
**Preschoolers’ self-report**								
Configural	284.73	240	–	0.032 (0.012–0.046)	0.985	–	0.981	–
Metric	305.19	252	20.46	0.034 (0.017–0.047)	0.982	0.003	0.978	0.003
Scalar	325.30	270	20.11	0.034 (0.017–0.046)	0.981	0.001	0.979	0.002
Factor variances	406.14	281	80.84	0.049 (0.038–0.060)	0.958	0.023	0.954	0.027
Residual variances	437.56	299	31.42	0.050 (0.040–0.060)	0.953	0.005	0.952	0.002
**Teachers’ ratings**								
Configural	391.97	240	–	0.081 (0.066–0.095)	0.927	–	0.907	–
Metric	402.43	252	10.46	0.079 (0.064–0.093)	0.928	0.001	0.912	0.005
Scalar	437.84	270	35.41	0.080 (0.066–0.094)	0.919	0.009	0.908	0.004
Factor variances	485.19	281	47.35	0.087 (0.074–0.100)	0.902	0.017	0.893	0.015
Residual variances	496.73	299	11.54	0.083 (0.070–0.095)	0.905	0.003	0.903	0.010

Analyzing of data rated by the preschoolers’ teachers showed that all CFIs and TLIs were below the cutoff value of 0.95, and RMSEAs were also above 0.06 in all models. The ΔCFI was ≤0.01 and ΔTLI ≤ 0.02, calculated by comparing the metric model to the configural model. Similarly, scalar invariance was also achieved because the ΔCFI ≤ 0.01 and ΔTLI ≤ 0.02 when the scalar model was compared to the metric model. However, invariance of factor variances was only partially supported because the ΔCFI > 0.01 while ΔTLI ≤ 0.02 when the factor variances model was compared to the scalar model. As a result, the scalar model was considered the final model, which represented that it hold identical patterns of factors, invariant factor loadings and intercepts of items, and factor means could be compared across gender (Byrne, 2004).

## Discussion and Implications

The main purpose of this study was to develop and validate an instrument for measuring and identifying preschoolers’ achievement goals, and to compare the effectiveness and efficiency of measurements rated by teachers with those reported by preschoolers themselves. The results indicated that the 6-factor achievement goal model was the best model in both samples when it was compared to the alternative models. The data reported by preschoolers themselves fitted fairly well, while that rated by teachers only reached an acceptable level. Moreover, the convergent and discriminant validity of the measurement of the former was good, while that of the latter was not good enough. Examinations of measurement invariance for both samples demonstrated that the 6-factor models hold identical patterns of factors, invariant factor loadings, and intercepts of items, which indicated that the model could be compared across gender. However, there is only partly identical factor means could be confirmed for the teacher sample because the indices of the 6-factor achievement goal model met the criteria in general, while they did not achieve an acceptable level in the preschooler sample.

The results clearly indicated that preschoolers as young as 5 years were already capable of evaluating their competence based, respectively, on an absolute standard (i.e., the mastery requirement of the task), an intrapersonal standard (i.e., their achievement in the past), or an interpersonal standard (i.e., others’ achievement); therefore, they approached potential positive outcomes (i.e., achieving the mastery requirement of the task) and avoided potential negative outcomes (i.e., their performance is considered inferior to that of others’). The present findings support the indirect evidence obtained from previous experimental research studies and qualitative observations, which suggested that preschoolers may either devote themselves to accomplishing their work or withdraw from work to avoid failure or being unable to complete their work (Rholes et al., 1980; Smiley and Dweek, 1994; Cimpian, 2017). Moreover, both personal performance and that of others’ in the past may be taken as standards against which preschoolers evaluate their current competence. This finding is in line with researchers’ practical and empirical observations/interviews which did not directly focus on investigating preschoolers’ achievement goals (Stipek and Hoffman, 1980; Mosatche and Bragonier, 1981; Pomerantz et al., 1995; Butler, 1998; Ihmeideh, 2015; Lapan and Boseovski, 2017).

The two measurement formats adopted the same text content in items used for preschoolers’ self-reporting and teachers’ ratings, respectively. Analysis of both samples indicated that the goodness of fit of the 6-factor achievement goal model was above an acceptable level. Only the results drawn from preschoolers’ self-reporting resulted in excellent construct validity, internal quality of model structure, and completely reached the requirement of scalar measurement invariance. This meant that the pictorial achievement goal measurement reported by preschoolers is valid and more efficacious than the pure text measurement rated by teachers. The findings indicated that the latter was more efficient than the former, because teachers’ ratings may take 6–7 min per child to complete on average, while preschoolers’ self-reporting may take 20–40 min per child due to larger individual differences. In addition, the pictorial items demonstrated that the content had the same meaning for boys and girls when the gender of the protagonist and the supporting role were the same as the preschool respondents. This implies that gender may be not the factor that affects measurement invariance. Preschoolers may also be attracted to the pictorial format as they are to hearing the story read out load, and children as young as 5 years can understand content representing their daily dialogue and behavior.

According to information processing theory, people’s attention can be attracted by preferred things, meaning that information (item content) is successfully sent into the short-term memory ready for working memory to process and respond to. Familiar information (i.e., content in the pictorial items representing preschoolers’ daily dialogue and/or behaviors) contributed to information retrieval from their long-term memory (i.e., preschoolers may search for such instances in their memory) to their working memory in order to respond to the questions posed. By evaluating the correspondence between external information and internal retrieved information, the preschoolers were able to choose one from the four options to represent the extent to which they are like or unlike the characters presented. In addition, current findings may be also implied that children as young as 5 years old have already developed sophisticated mental imagery process. There were four mental imagery processes: generation, maintenance, inspection, and transformation. Preschoolers were enabled to maintain the images, which were used in this study as pictorial measurement, in their short-term memory, and to scan images and to activate the images or information about themselves in long-term memory for checking their similarity (Guarnera et al., 2017, 2019). There may be at least three mental imagery process involving in the measurement process. It suggests that the corresponding mental imagery process underlying the implementation of this pictorial achievement goal measurement may be also worth further investigating.

Finally, rating by others used in previous achievement motivation research may not be the most suitable method because the achievement goals may not be accurately operationalized, and preschoolers were capable of reporting themselves precisely by using current pictorial items. This suggests that future studies could adopt this pictorial achievement goal measurement to investigate preschoolers’ achievement motivation rather than using a manipulated task rated by others. However, solution should be devised to overcome its limitation regarding efficiency.

## Summary

Preschoolers’ achievement goals and related issues have never been directly investigated; only a few research studies have conducted experiments to indirectly examine achievement goals through analyzing the ratings of others’. This may reflect an underestimation of preschoolers’ abilities to self-evaluate and self-report their achievement goals and/or that there were several difficulties in the design of measurements. By examining evidence from experimental and qualitative research, this study proposed that preschoolers may be capable of evaluating and reporting on themselves according to three different referents, and to approach positive and avoid negative possibilities. Through intensive observation, familiar scenes regarding preschoolers’ daily dialogue and behaviors represented by the pictorial format were developed to be attractive to young children and facilitate information retrieval so that preschoolers could successfully respond to the items. This format proved effective for measuring preschoolers’ achievement goals, and it was confirmed that the items had equal meanings for boys and girls, but that the pictorial measurement has room for improvement regarding efficiency. In contrast, the pure text format used for teachers to rate preschoolers’ achievement goals had only at an acceptable level of effectiveness; the factor variances invariance across gender did not compensate for this limitation despite it being much more efficient than the pictorial format used for self-reporting by the preschoolers themselves.

## Limitations of the Study

The limitations of the study center around the nature of the sample, and the effectiveness and efficiency of the measurement formats: pictorial and text items measurement formats. First, because a nonrandom stratified sample was used and this was a preliminary study of preschoolers’ achievement goals, the effectiveness of pictorial achievement goal measurement based on a 6-factor model for understanding 5-year-old preschoolers’ achievement goals may not necessarily be suitable for other samples of the same age or below this age. Second, although the pictorial measurement is equally effective for measuring 5-year-old preschooler boys’ and girls’ achievement goals, the implementation process is time-consuming, which may therefore limit its implementation on a large scale. Third, despite text measurements being conducted by the preschoolers’ teachers, the validity was not good enough for implementation in future studies conducted to accurately assess preschoolers’ achievement goals. As a result, the efficiency of teachers’ ratings may not compensate for its limitation regarding effectiveness. Future researches adopt either format to investigate related issues, the tradeoff between effectiveness and efficiency in measuring achievement goals should be considered. It is also recommended that solutions are proposed to improve both the efficiency of pictorial self-reporting measurement and the effectiveness of the pure text format for teachers’ ratings.

## Data Availability Statement

The original contributions presented in the study are included in the article, further inquiries can be directed to the corresponding author.

## Ethics Statement

The studies involving human participants were reviewed and approved by National Cheng Kung Human Subjects Institutional Review Board. Written informed consent to participate in this study was provided by the participants’ legal guardian/next of kin.

## Author Contributions

The author confirms being the sole contributor of this work and has approved it for publication.

## Funding

This study was funded by the Taiwan Ministry of Science and Technology (grant nos. 109-2511-H-153 -004 -MY2 and 109-2410-H-153-015-).

## Conflict of Interest

The author declares that the research was conducted in the absence of any commercial or financial relationships that could be construed as a potential conflict of interest.

## Publisher’s Note

All claims expressed in this article are solely those of the authors and do not necessarily represent those of their affiliated organizations, or those of the publisher, the editors and the reviewers. Any product that may be evaluated in this article, or claim that may be made by its manufacturer, is not guaranteed or endorsed by the publisher.
